# Pentacle gold–copper alloy nanocrystals: a new system for entering male germ cells *in vitro* and *in vivo*

**DOI:** 10.1038/srep39592

**Published:** 2016-12-21

**Authors:** Yu Lin, Rong He, Liping Sun, Yushan Yang, Wenqing Li, Fei Sun

**Affiliations:** 1 School of Life Sciences, University of Science and Technology of China, Hefei, Anhui 230027, P. R. China; 2 Hefei National Laboratory for Physical Sciences at the Microscale, Center of Advanced Nanocatalysis (CAN-USTC), Department of Chemical Physics, University of Science and Technology of China, Hefei, Anhui 230026, P. R. China; 3 Department of Pathophysiology, Basic Medical College, Anhui Medical University, Hefei, Anhui 230032, P. R. China

## Abstract

Gold-based nanocrystals have attracted considerable attention for drug delivery and biological applications due to their distinct shapes. However, overcoming biological barriers is a hard and inevitable problem, which restricts medical applications of nanomaterials *in vivo*. Seeking for an efficient transportation to penetrate biological barriers is a common need. There are three barriers: blood-testis barrier, blood-placenta barrier, and blood-brain barrier. Here, we pay close attention to the blood-testis barrier. We found that the pentacle gold–copper alloy nanocrystals not only could enter GC-2 cells *in vitro* in a short time, but also could overcome the blood–testis barrier and enter male germ cells *in vivo*. Furthermore, we demonstrated that the entrance efficiency would become much higher in the development stages. The results also suggested that the pentacle gold–copper alloy nanocrystals could easier enter to germ cells in the pathological condition. This system could be a new method for theranostics in the reproductive system.

Gold-based nanocrystals have been widely used in drug delivery and other biological applications due to their advantageous biocompatibility, plasmonic properties^1^,^2^,^3^,^4^,^5^,^6^. However, biological barriers are the stumbling blocks to nanomaterials in biomedical applications^7^,^8^,^9^. Therefore, searching for a successful delivery system to achieve fast and efficient transportation to penetrate biological barriers is the only way which must be passed. Our previous work reported the synthesis of pentacle gold–copper nanocrystals as a new class of gold-based nanostructures with their low cost of alloying and high photothermal performance^10^. Using a remarkably simple co-reduction synthetic route, gold and copper precursors can be made to generate a starfish-like nanostructure with five long branches, which are used for tumor photothermal therapy. Because of their sharp arms, the pentacle nanocrystals may find distinctive use in applications related to penetrating some biological barriers.

Blood–testis barrier, blood–placenta barrier, and blood–brain barrier are three biological barriers which prevent nanomaterial applications in medical fields^11^. Here, we investigated the blood–testis barrier. Spermatogenesis in the testes is a coordinated process, which includes spermatogonial stem cell division into spermatogonia, and diploid spermatogonia differentiate into meiotic spermatocytes, which divide twice without additional DNA replication, producing haploid round spermatids^12^,^13^,^14^,^15^,^16^. Only two cell types constitute the seminiferous epithelium, the Sertoli and the germ cells. Spermatogenesis occurs in a very safe environment because of the blood–testis barrier (BTB), which is formed by adjacent Sertoli cells at the basal compartment of the seminiferous epithelium^17^,^18^,^19^,^20^,^21^,^22^. In theory, nanoparticles cannot penetrate the BTB, which prevents nanoparticles producing toxicity in male germ cells^17^. However, several studies have shown that nanoparticles can penetrate the BTB while non-nanoparticles can not^17^. In recent years, some reports have indicated biodistribution of gold nanoparticles in the testes in both time-dependent and size-dependent manners^23^,^24^. The previous reports indicate that route of administration, dose of AuNP and AuNP characteristics (e.g., size, shape, chemical composition and surface charge) play a significant role in determining the reproductive nanotoxicity of AuNP on spermatogenesis^25^,^26^. The shape of AuNP is an important characteristic, which may determinate the biodistribution or biotoxicity on spermatogenesis. Our previous work reported pentacle gold-copper nanocrystals were a potent agent for tumor therapy^10^. Therefore, it is important and necessary to study the biocompatibility, biological effects and biomedical application of the pentacle gold-copper alloy nanocrystals deeply. However, to our knowledge, no other reports have been published about testicular biodistribution or bioapplication of pentacle gold–copper alloy nanocrystals.

Herein, we have found that pentacle gold–copper alloy nanocrystals entered GC-2 cells (a spermatocyte cell line) within a short time *in vitro*, and showed the ability of pentacle nanocrystals to penetrate the BTB *in vivo*. We have also investigated a way to increase the amount of pentacles in the testes. In addition, the pentacles were combined with fluorescein isothiocyanate isomer I (FITC) as a nanocomplex model to assess their potential for carrying siRNA/miRNA/DNA or polypeptides into male germ cells *in vivo* to solve issues of male infertility.

## Results

### Pentacle gold–copper alloy nanocrystals enter GC-2 cells *in vitro* without cytotoxicity

**Figure 1:**
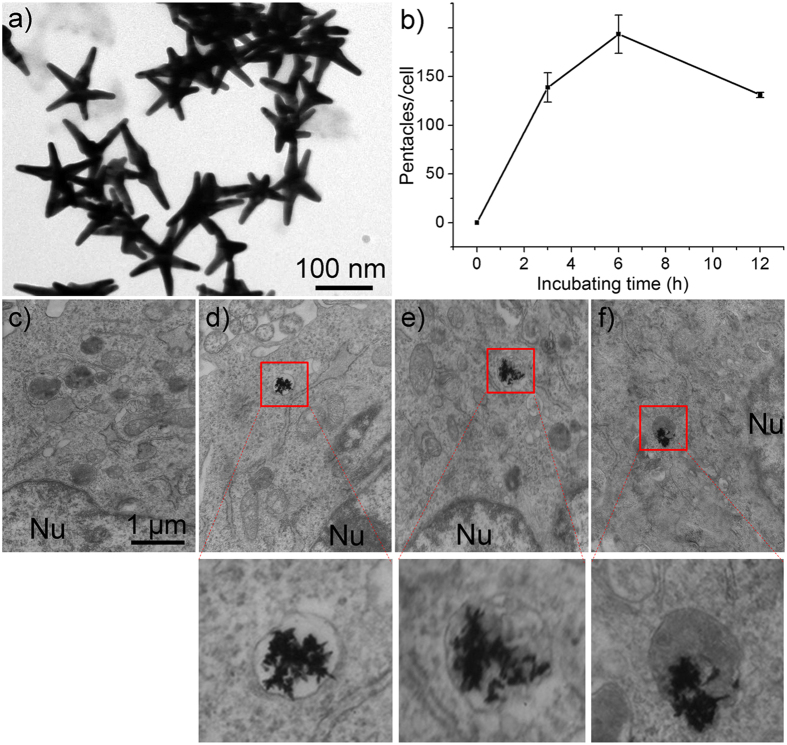
The cellular uptake of PEG-modified pentacle gold–copper alloy nanocrystals (pentacle NCs) by GC-2 cells. (**a**) TEM images of PEG-modified pentacles. (**b**) The number of pentacles (calculated by ICP-MS) per cell at different incubation times for treating GC-2 cells with 15 μg mL^−1^ PEG-pentacles. (**c**–**f**) TEM images of the endocytosis of PEG-pentacles into GC-2 cells at 0, 3, 6 and 12 h, respectively. Nu indicates the cell nucleus.

Pentacle gold–copper alloy nanocrystals were synthesized through a modified strategy in our previous study in methods^10^. Figure 1a shows a transmission electron microscope (TEM) image of pentacles, indicating that most of the nanocrystals are five-fold twined structures with sharp arms. To stabilize the structure and prolong blood circulation, we decorated the pentacles with thiolated poly(ethylene glycol) (PEG) for future use.

Many studies have demonstrated that the uptake of gold nanoparticles by mammalian cells is size- and shape-dependent^27^,^28^,^29^,^30^. To investigate the uptake of pentacles in mammalian reproductive cells, the as-synthesized pentacle-PEG was added to GC-2 cells. After incubating for different times, the accumulation of pentacle-PEG in GC-2 cells was analyzed by inductively coupled plasma mass spectrometry (ICP-MS) (Fig. 1b). The results show that PEG-modified pentacle gold–copper alloy nanocrystals entered GC-2 cells after a short time. The maximum uptake appeared at 6 h, and then the amount of pentacles decreased about 30% at 12 h. Also, we found pentacles after 3 h in TEM images, which further demonstrated that pentacle-PEG entered GC-2 cells within a short time (Fig. 1c–f).

**Figure 2:**
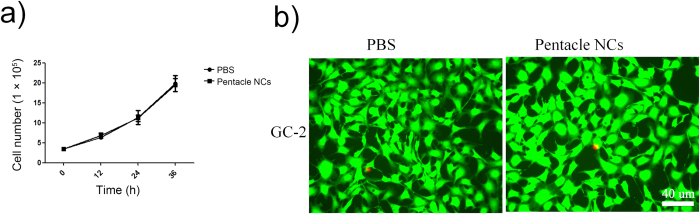
The cytotoxicity of PEG-modified pentacle gold–copper alloy nanocrystals (pentacle NCs) to GC-2 cells. (**a**) Cell viabilities of GC-2 cells treated with pentacle NCs for different incubation times were determined by standard MTT assay. GC-2 cells treated with PBS were used for the control group. (**b**) Fluorescent staining of GC-2 cells with calcein-AM for living cells (green fluorescence) and with the nuclear stain propidium iodide (PI) for dead cells (red fluorescence). PBS-treated cells were used as a control group.

The cytotoxicity of pentacle gold–copper alloy nanocrystals of different concentrations to the GC-2 cell line was investigated. We found there were no significant cytotoxicity at 15, 30, 60 μg ml^−1^ pentacle NCs after 24 h (Supplementary data, Figure S1). The pentacle-PEG did not appear to be toxic at 15 μg mL^−1^ (gold atom concentration) measured by a standard MTT assay (Fig. 2a) at 12, 24 and 36 h. Also, we double stained GC-2 cells with calcein-AM for living cells and with the nuclear stain propidium iodide (PI) as an indicator of dead cells. There was no significant difference in cell death between pentacle-PEG treatment and the phosphate buffer solution (PBS) group (Fig. 2b). These results suggest that the pentacles do not have an impact on cell proliferation and death, indicating good biocompatibility with mammal reproductive cells *in vitro*.

**Figure 3:**
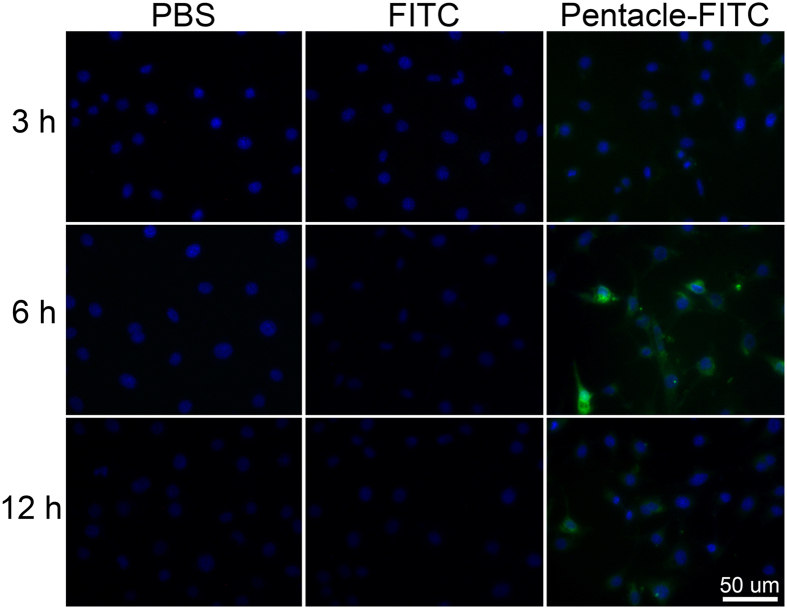
Green fluorescence images of GC-2 cells at different times. We used FITC-labeled pentacle gold–copper alloy nanocrystals (pentacle-FITC) as a nanocomplex model and exposed pentacle-FITC to GC-2 cells for 3, 6 and 12 h. Nuclei (blue fluorescence) were stained with Hoechst 33342. In the negative group, we treated GC-2 cells with PBS, and in the control group, we treated GC-2 cells with free FITC.

To simulate a nanocomplex system, we labeled pentacles with FITC. We exposed FITC-labeled pentacles to GC-2 cells for 0, 3, 6 and 12 h. In contrast, the same concentration of FITC was also incubated with GC-2 cells. Figure 3 shows the results of fluorescence emission detection. There was no green fluorescence except for the pentacle-FITC group, which demonstrated that the pentacles could deliver FITC molecules to GC-2 cells efficiently. In the pentacle-FITC group, the quasi-round structures with green fluorescence were assigned as endocytic vesicles which took pentacles to the cytomembrane of GC-2 cells. Consistent with the ICP-MS results in Fig. 1b, the highest fluorescent signal in the pentacle-FITC group appeared at 6 h treatment, further demonstrating that the pentacles entering GC-2 cells was a fast procedure. The results suggested the pentacles could deliver biological molecules efficiently to GC-2 cells *in vitro*.

### Pentacle gold–copper alloy nanocrystals enter male germ cells of adult mouse *in vivo*

**Figure 4:**
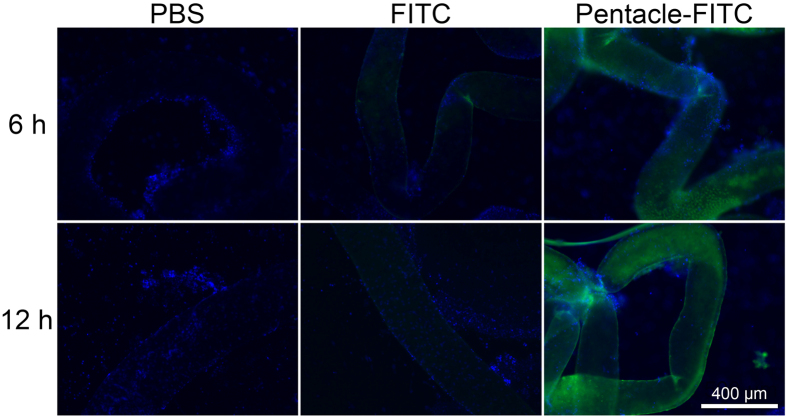
Green fluorescence images of the seminiferous tubules of mouse testes. PBS, free FITC or pentacle-FITC was injected directly into the mouse testes; 6 and 12 h after injection, the green fluorescence images of seminiferous tubules of different groups were taken by fluorescence microscopy. Nuclei (blue fluorescence) were stained with Hoechst 33342.

Although pentacles can be taken up by GC-2 cells *in vitro*, it is also possible for the BTB to prevent pentacles entering male germ cells *in vivo*. It is an important issue to study whether the pentacle gold–copper alloy nanocrystals can penetrate the BTB as expected. In the present study, we firstly used FITC-labeled pentacles to investigate whether they can penetrate the BTB with the conjunct organic molecules. We directly injected PBS, free FITC or FITC-labeled pentacles into mouse testes *in situ*, which meant it was injected into the interspace of the seminiferous tubules outside the BTB. We collected testes of adult male mice and stretched the seminiferous tubules on the slide at each time point. As shown in Fig. 4, the fluorescence images in the FITC group are similar to that of the PBS group, indicating that almost all the free FITC molecules are blocked by the BTB. In contrast, the green fluorescent signal of the pentacle-FITC group was high enough to fill the overall structure of the seminiferous tubules in the chosen images. The results demonstrated that the pentacles could pass through the BTB.

**Figure 5:**
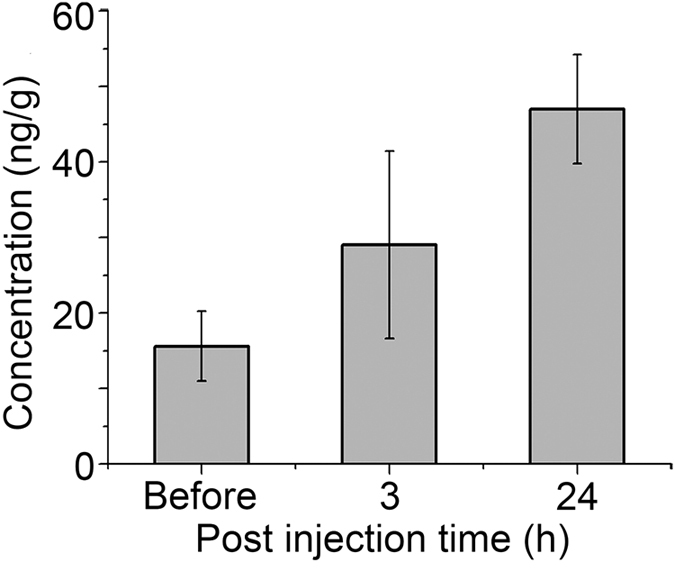
Distribution of PEG-modified pentacle gold–copper alloy nanocrystals (pentacle NCs) in adult mouse testes. ICP-MS analysis of metal contents in the testes of adult mice at different times. Results are mean ± SD of eight animals in each group.

**Figure 6:**
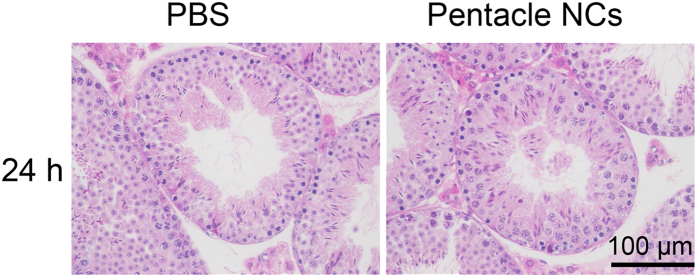
Morphological analysis of mouse testes with pentacle NC treatment. Testicular cross-sections from controls and pentacle NC-treated mice were stained with H&E.

We also carried out the experiment by intravenous (i.v.) injection to male mice. PBS and pentacle-PEG were injected via the tail vein once, respectively. 3 and 24 h after injection, the accumulation of pentacles in testes was analyzed by ICP-MS. The results show that approximately 2.5 and 6.6 ng of pentacle nanocrystals were found in the testes 3 and 24 h after i.v. injection, respectively (Fig. 5). Although the amount of pentacles in the testes was small, the increasing trend suggests that, with repeated exposure, more pentacle nanocrystals would be expected to accumulate in the testes. They could potentially pass through the BTB. Also, the male reproductive toxicity was investigated; as shown in Fig. 6, there were no significant differences in testicular morphology between pentacle-treated mice and the control group. All the results suggest that the pentacles could penetrate the BTB and have good biocompatibility *in vivo*.

We have proven that the pentacle nanocrystals can pass through the BTB, but the BTB prevents more pentacles entering the seminiferous epithelium and entering the male germ cells. The results still show that the amount of pentacle nanocrystals in the testes was small, which was consistent with other reported results^26^,^31^. Therefore, methods and conditions for increasing the amount of pentacles passing the BTB should be investigated.

### In puberty or pathological conditions, increasing the amount of pentacles gold–copper alloy nanocrystals in the testes

**Figure 7:**
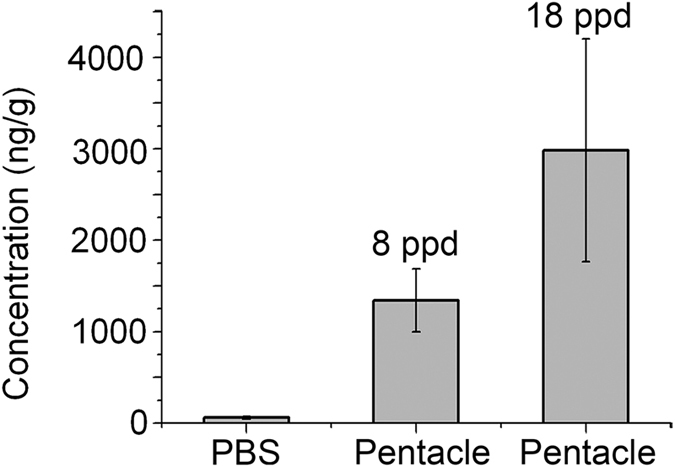
Distribution of PEG-modified pentacle gold–copper alloy nanocrystals (pentacle NCs) in puberty rat testes. ICP-MS analysis of metal contents in the testes of 8 and 18 ppd SD rats. In each group, animals were treated with a single i.p. administration of pentacle NCs for 24 h. Results are mean ± SD of eight animals in each group.

During development stages, the BTB is gradually formed by Sertoli cells. The BTB has been reported to be established in rats by 15–18 days postpartum (ppd)^32^,^33^. In puberty, harmful substances in the environment can more easily enter germ cells. In the present study, 8 and 18 ppd rats were intraperitoneally (i.p.) injected with PBS or PEG-modified pentacle gold–copper alloy nanocrystals in a single dose. The accumulation of pentacle nanocrystals in the testes was analyzed by ICP-MS. As shown in Fig. 7, approximately 1.35 ng mg^−1^ (Au–Cu/testis weight) and 2.97 ng mg^−1^ of pentacles were found in the testes 24 h after i.p. injection, respectively. It is about 50 ng/g (Au–Cu/testis weight) in adult mice testes (Fig. 5), which was above 2000 ng/g (Au–Cu/testis weight) in puberty rat testes (Fig. 7). These results indicate that the pentacle nanocrystals can enter the male germ cells in large amounts in juvenile rats. Also, we found branched nanocrystals in spermatocytes of 8 ppd rats from the TEM image (Supplementary data, Figure S2), resembling the arms of a whole pentacle. In puberty, the pentacles can enter germ cells in large amounts in normal physiological conditions, which suggests that we can use the pentacles as a delivery system for intervening in and treating the diseases of male germ cells *in vivo*.

The BTB serves as a “gate-keeper” that regulates proteins and/or biomolecules that can enter the adluminal compartment from the interstitium^34^,^35^. However, the BTB is not an impervious barrier. During normal physiological conditions of murine spermatogenesis, preleptotene/leptotene spermatocytes transit and migrate from the basal to the adluminal compartment through the BTB during stages VIII–IX, in which the BTB has transitory permeability^36^,^37^,^38^,^39^. In some abnormal conditions, many materials and treatments such as adjudin, cadmium, and hot baths can also destroy the BTB and enhance its permeability^40^,^41^,^42^,^43^,^44^,^45^,^46^. For example, when treated in a hot bath, the BTB will be destroyed and exogenous materials will enter germ cells more easily. In the present study, we treated the mice at 43 °C for 10 min to mimic the pathological condition cryptorchidism, which destroyed the BTB; 24 h after hot bath treatment, we directly injected PBS, free FITC or FITC-labeled pentacle gold–copper alloy nanocrystals into mouse testes *in situ*. The fluorescence images show that the fluorescent signal of FITC-labeled pentacle gold–copper alloy nanocrystals within hot bath treatment was much higher than in groups at room temperature treatment (Supplementary data, Figure S3). The results suggest that the pentacle gold–copper alloy nanocrystals can more easily enter germ cells in pathological conditions. Therefore, if the BTB has been formed stably, we can use pentacles as delivery vehicles and cure the diseases of male germ cells under heat treatment *in vivo*.

## Discussion

Through shape control, the synergy of combining gold and copper metals can be better exploited. We have reported an aqueous phase route to the synthesis of pentacle gold–copper alloy nanocrystals with five fold twinning, the size of which can be tuned in the range from 45 to 200 nm. The pentacle gold–copper alloy nanocrystals showed plasmonic and catalytic properties, and had notable photothermal effect to tumours in our previous study. In the present, we investigated whether the pentacle gold–copper alloy nanocrystals cause harmful impact on the male reproductive system. We exploited pentacle gold–copper alloy nanocrystals to enter GC-2 cells *in vitro* and penetrate the BTB *in vivo*. We demonstrated that the PEG-modified pentacle gold–copper alloy nanocrystals can enter GC-2 cells within a short time, and have good biocompatibility with GC-2 cells *in vitro*. Also, we proved that the pentacle gold–copper alloy nanocrystals can pass through the BTB in adult male mice, and found ways to improve the efficiency of entrance to the testes. In order to simulate delivery circumstances, FITC-labeled pentacle nanocrystals were also involved in the research. We further found that the pentacle nanocrystals can enter male germ cells in large amounts in juvenile rats, suggesting that we can deliver drugs or biomolecules by the nanoparticles at this stage. For example, some genetic factors can cause infertility. We can deliver the genes and siRNA or CRISPR/Cas9 (a gene editing system) into the testis through destroying or re-establishing the BTB at the puberty. Our results could provide a method for high-branched nanocrystals to be applied to theranostics in the genital system.

## Methods

### Synthesis of pentacle gold–copper alloy nanocrystals

In a standard synthesis of pentacle gold–copper alloy nanocrystals, 0.33 mL of aqueous CuCl_2_·2H_2_O (100 mM), 0.27 mL of aqueous HAuCl_4_·3H_2_O (100 mM), 45 mg of HDA, 1.4 mL of aqueous glucose (1 M) and 3 mL of water were added to a 20 mL vial at room temperature. After the vial had been capped, the solution was magnetically stirred at room temperature overnight. The capped vial was then transferred into an oil bath and heated at 100 °C for 15 min under magnetic stirring. As the reaction proceeded, the solution changed its color from Kelly green to brown. To prepare samples for electron microscopy characterization, the pentacle nanocrystals were centrifuged at 10,000 rpm for 8 min and washed by water three times and ethanol twice to remove excess precursor, HDA and glucose.

### PEG and FITC modified pentacles

In a typical process, 10 mg of HS-PEG-NH_2_ was added to an aqueous suspension of Au-Cu pentacles (1 mg/mL, 5 mL). The reaction mixture was vortexed immediately and then magnetic stirred overnight, followed by centrifuging at 14000 rpm for 5 min to remove excess PEG. For FITC-pentacles, 0.1 mL of 1 mM FITC solution was added to 5 mL of HS-PEG-NH_2_ functionalized pentacle solution. The mixture was magnetic stirred for several hours. Then the final solution was centrifuged at 10,000 rpm for 8 min and washed by water three times to remove dissociative FITC.

### Cell culture

GC-2 cells were grown in Dulbecco’s modified Eagle’s medium supplemented with 10% fetal bovine serum (Life Technologies Inc.) and 1% antibiotics (100 Units mL^−1^ penicillin and 100 mg mL^−1^ streptomycin; Life Technologies Inc.) and cultured at 37 °C with 5% CO_2_.

### Animals

Animals were handled according to the guidelines of experimental animal ethics committee of Anhui medical university, and the study was reviewed and approved by the experimental animal ethics committee of Anhui medical university.

### ICP-MS

*In v*itro, the 6-well plate GC-2 cells were exposed to PEG-modified 100 nm pentacle gold–copper alloy nanocrystals at 15 μg mL^−1^ for 0, 3, 6 and 12 h. At each time point, the cells were counted, collected and dissolved in aqua regia. The amount of gold in the testes was measured by ICP-MS (Atomscan Advantage, Thermo Jarrell Ash Corporation, USA). We used a simple model to calculate the pentacle nanocrystals by measured the ICP-MS results^10^. A pentacle contains a decahedron and ten tetrahedra. Considering the average edge length of pentacle nanocrystals in the TEM image, the volume of a pentacle (*V*) can be calculated to be 26200 nm^3^. According to our previous work, the pentacle nanocrystals were indexed as typical face-centered cubic (*fcc*) structure. Hence a cell contains 4 atoms. The volume of a unit cell (*V*_0_) was calculated to be 0.064 nm^3^. Therefore, each pentacle contains *n*_0_ = 4 × *V*/*V*_0_ = 1.64 × 10^6^ atoms. The average relative atomic mass (*M*) of the Au-Cu alloy pentacles was 178 (Au 86%, Cu 14%). Meanwhile, the total mass of the Au and Cu metals (*m*) was determined by ICP-MS. The total number of atoms (*n*) was *m*/*M* * *N*_*A*_, where N_A_ represents Avogadro constant.

*In vivo*, for ICP-MS analyses, 6–8 week-old mice were i.v. injected with PBS or 12.5 mg kg^−1^ (by body weight) PEG-modified 100 nm pentacle gold–copper alloy nanocrystals once (single dose); 3 and 24 h after injection, the testes were collected, weighed and then dissolved in aqua regia. The amount of gold in the testes was measured by ICP-MS (Atomscan Advantage, Thermo Jarrell Ash Corporation, USA).

*In vivo*, for ICP-MS analyses, 8 and 18 ppd rats were i.p. injected with PBS or 25 mg kg^−1^ (by body weight) PEG-modified 100 nm pentacle gold–copper alloy nanocrystals once (single dose); 24 h after injection, the testes were collected, weighed and then dissolved in aqua regia. The amount of gold in the testes was measured by ICP-MS.

### TEM

*In vitro*, the GC-2 cells were exposed to PEG-modified 100 nm pentacle gold–copper alloy nanocrystals at 15 μg mL^−1^ for 0, 3, 6 and 12 h. At each time point, the cells were collected, then centrifuged, fixed with 2.5% glutaraldehyde solution for 6 h, dehydrated through an ethanol series (70% for 15 min, 90% for 15 min, and 100% for 15 min twice) and embedded in Epon/Araldite resin (polymerization at 65 °C for 15 h). Thin sections were cut (70 nm thick), placed on grids and stained for 1 min each with 4% uranyl acetate (1:1, acetone/water) and 0.2% Reynolds lead citrate (water), air-dried and imaged under an 80 kV JEOL-1230 TEM.

*In vivo*, 8 and 18 ppd SD rats were i.p. injected with PBS or 25 mg kg^−1^ (by body weight) PEG-modified 100 nm pentacle gold–copper alloy nanocrystals once (single dose); 24 h after injection, the testes were collected and fixed with 2.5% glutaraldehyde solution for 6 h. Finally, they were imaged under an 80 kV JEOL-1230 TEM.

### Fluorescence microscopy

*In vitro*, the GC-2 cells were exposed to FITC-labeled 100 nm pentacle gold–copper alloy nanocrystals at 15 μg mL^−1^ for 0, 3, 6 and 12 h. In the negative group, we treated GC-2 cells with PBS, and in the control group, we treated GC-2 cells with free FITC. The cells were washed twice with ice-cold PBS and fixed with 4% paraformaldehyde for 20 min. Nuclei were stained with Hoechst 33342 (Sigma). Fluorescent signals were examined using a Nikon Eclipse 80i epifluorescence microscope.

*In vivo*, PBS, free FITC or 12.5 mg kg^−1^ (by body weight) FITC-labeled 100 nm pentacle gold–copper alloy nanocrystals were injected directly into mouse testes; 6 and 12 h after injection, the testes were cut, put on the slides and then the seminiferous tubules were pulled by tweezers. The seminiferous tubules were stained with Hoechst 33342 (Sigma) and covered by a cover glass. Fluorescent signals were examined using a Nikon Eclipse 80i epifluorescence microscope.

### Statistical analysis

Student’s t-test was applied to examine the differences among variables. Data are shown as mean ± SD, unless otherwise indicated. *p* values ≤0.05 were considered to be statistically significant.

## Additional Information

**How to cite this article**: Lin, Y. *et al*. Pentacle gold–copper alloy nanocrystals: a new system for entering male germ cells *in vitro* and *in vivo. Sci. Rep.* **6**, 39592; doi: 10.1038/srep39592 (2016).

**Publisher's note:** Springer Nature remains neutral with regard to jurisdictional claims in published maps and institutional affiliations.

## Supplementary Material

Supplementary Information
