# Supplementary material for: Pentacle gold–copper alloy nanocrystals: a new system for entering male germ cells in vitro and in vivo
Source: Sci Rep. 2016 Dec 21;6:39592. doi: 10.1038/srep39592 (PMC5175129; doi:10.1038/srep39592)
Supplement: Supplementary Information [file srep39592-s1.doc]

Supporting Information

**Pentacle Gold–Copper Alloy Nanocrystals: A New System for Entering Male Germ Cells *in vitro* and *in vivo***

Yu Lin, Rong He, Liping Sun, Yushan Yang, Wenqing Li, and Fei Sun


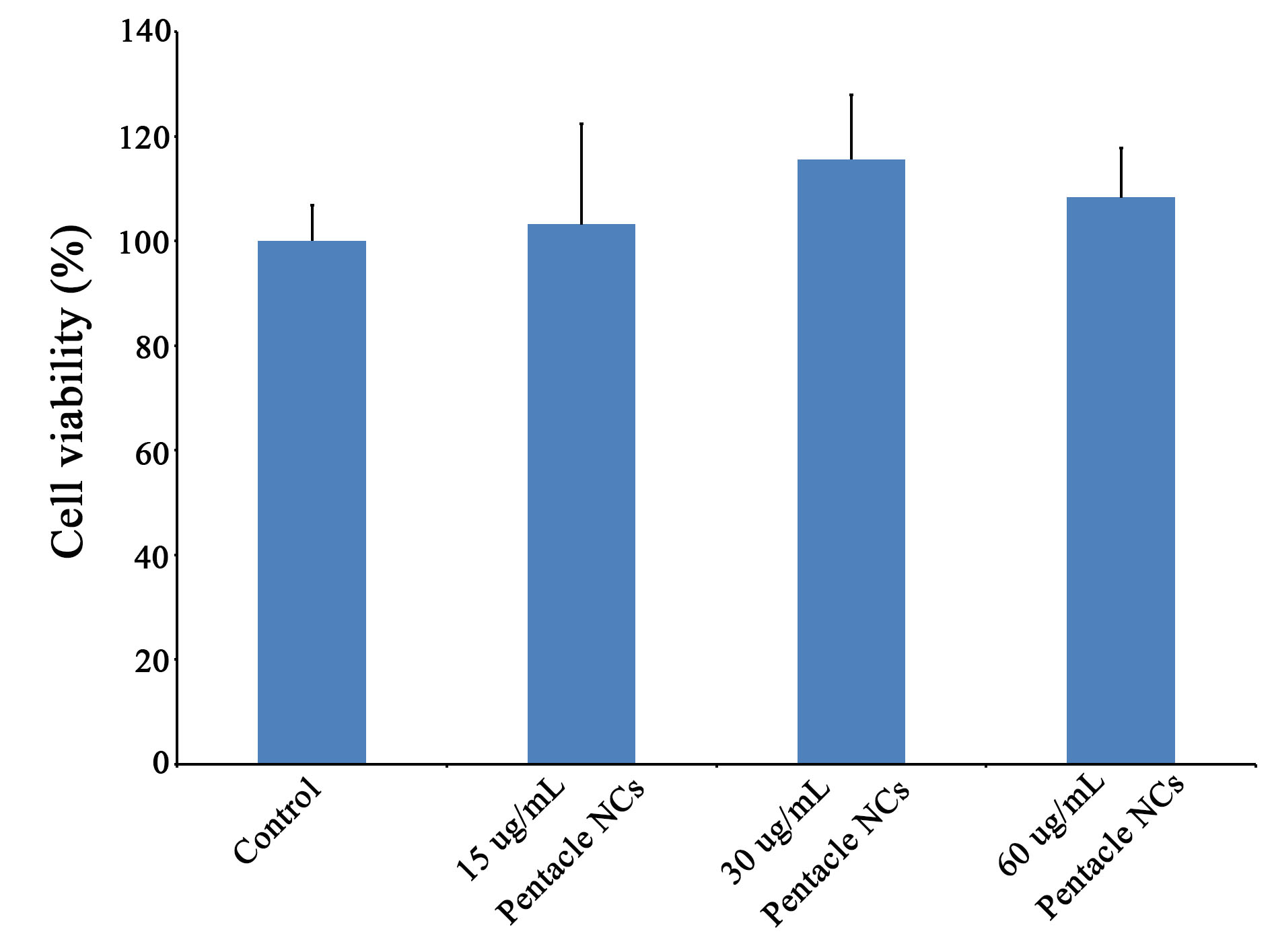


**Figure S1. The cell viability of PEG-modified pentacle gold–copper alloy nanocrystals (pentacle NCs) to GC-2 cells.** Cell viabilities of GC-2 cells treated with pentacle NCs at different concentrations were determined by standard MTT assay. GC-2 cells treated with PBS were used for the control group.


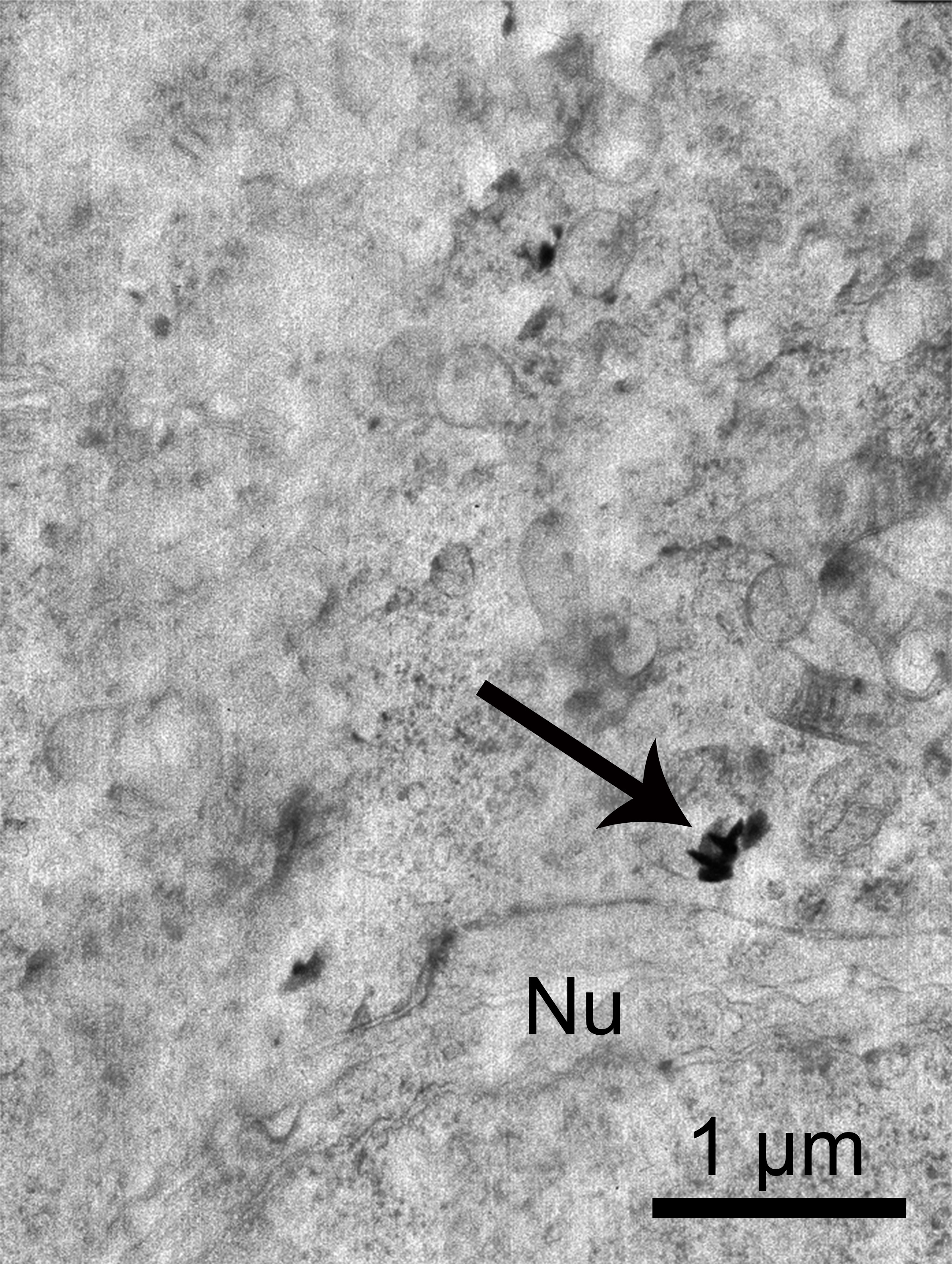


**Figure S2.** **TEM image of spermatogonial types in testis sections from pentacle-treated 8 ppd SD rats.** Black pentacles are pentacles.


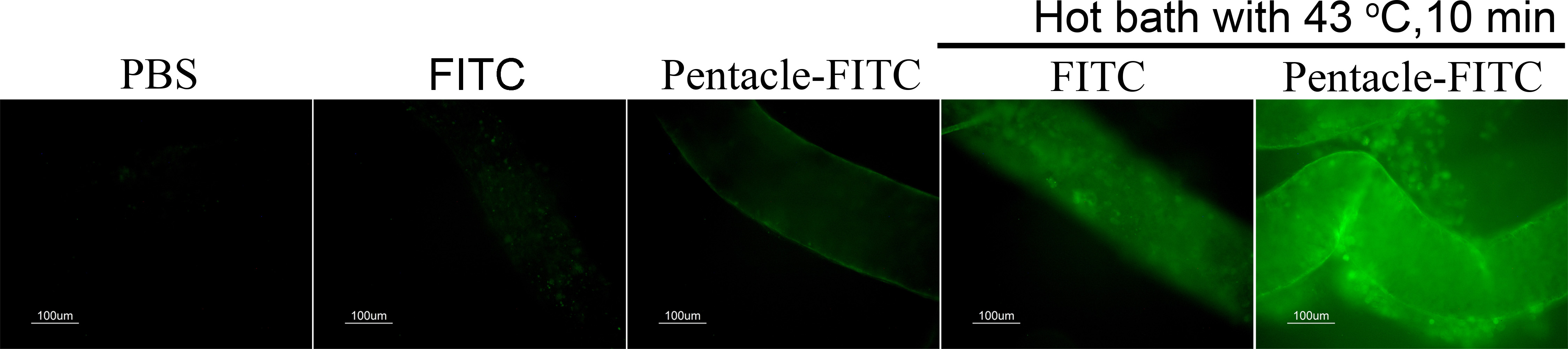


**Figure S3. Green fluorescence images of FITC-labeled pentacle gold–copper alloy nanocrystals (pentacle-FITC) in the seminiferous tubules of mouse testes.** Adult male ICR mice were treated at room temperature or at 43 °C for 10 min; after 24h, PBS, free FITC or pentacle-FITC was injected directly into the mouse testes for 4 h. Fluorescein emission in the seminiferous tubules was detected by fluorescence microscopy. The negative group was treated with PBS, and the control group was treated with free FITC.
